# The early predictive value of platelet-to-lymphocyte ratio to hemorrhagic transformation of young acute ischemic stroke

**DOI:** 10.2478/abm-2023-0069

**Published:** 2023-12-28

**Authors:** Huijun Wen, Ning Wang, Min Lv, Yue Yang, Hongmei Liu

**Affiliations:** Department of Neurology, Baoji Central Hospital, Baoji, Shaanxi 721008, China; Department of Rehabilitation Medicine, Baoji Traditional Chinese Medicine Hospital, Baoji, Shaanxi 721008, China; Department of Cardiology, Shangluo Beikuanping Central Hospital, Shangluo, Shaanxi 726000, China

**Keywords:** blood–brain barrier, cerebral hemorrhage, cerebral infarction, magnetic resonance imaging, platelet count

## Abstract

**Background:**

The increasing incidence of acute ischemic stroke (AIS) necessitates a comprehensive understanding of the related factors. Hemorrhagic transformation (HT), a severe complication of AIS, is influenced by platelet-induced inflammation and lymphocyte levels.

**Objective:**

To measure the predictive value of platelet-to-lymphocyte ratio (PLR) in the occurrence of HT in young AIS patients.

**Methods:**

Data of young AIS patients (n = 157) admitted to the hospital for the first time were retrospectively collected. The patients were divided into HT (63 patients) and non-HT groups (94 patients) on the basis of whether HT had occurred after admission. The National Institute of Health stroke scale (NIHSS) score was used to determine the severity of clinical symptoms. The relationship between PLR and HT and NIHSS scores was analyzed to evaluate the predictive value of PLR in the occurrence of HT using receiver operating characteristic (ROC) and area under the curve (AUC).

**Results:**

Multivariate analysis showed that PLR and NIHSS are independent risk factors of HT. The PLR value of the observation group was positively associated with the NIHSS score (*r* = 0.8075, *P* < 0.0001). According to the PLR prediction about the occurrence of HT, an AUC of 0.713 (95% CI, 0.652–0.781), a cut-off value of 109.073, and a sensitivity and specificity of 0.806 and 0.674, respectively, were obtained.

**Conclusions:**

PLR value can predict the possibility of HT in young AIS patients to a certain extent. To take effective measures to prevent HT in advance has crucial clinical significance according to PLR value.

With increasing social development and the pressure of daily life, the incidence of young acute ischemic stroke (AIS) is increasing year by year [1]. After occurrence of AIS in the young, in the middle segment of the blood flow of the diseased blood vessel, acute ischemia and hypoxia of the brain cells in the blood supply area break the balance between the internal and external nerves and blood vessels [2], resulting in destruction of the blood–brain barrier (BBB). This causes blood extravasation and local reperfusion injury, leading to hemorrhagic transformation (HT) [3]. HT refers to secondary bleeding after acute cerebral infusion (ACI). The incidence rate reported in the literature is 8.5%–30% [4]. Studies have shown that platelet-induced inflammation plays a vital role in the pathogenesis of ACI [5], and lymphocyte levels decrease because of various pathological changes during acute ischemic events [6]. Studies have reported that the platelet-to-lymphocyte ratio (PLR) is a simple, reproducible, easy-to-obtain parameter for rapid assessment of inflammatory response [7], wherein its increased levels will increase the risk of poor prognosis and infarct size in patients with ACI [8]. At present, few clinical reports exist on the relationship between PLR and HT in young AIS patients. In this study, cranial computed tomography (CT) or magnetic resonance imaging (MRI) was used to determine the presence of HT in young AIS patients. The National Institute of Health stroke scale (NIHSS) score was used to determine the severity of the patient's clinical symptoms, and blood routine and the related biochemical indicators were tested at the same time to explore the predictive value of PLR in the occurrence of HT in young AIS patients.

## Methods

We retrospectively collected the clinical medical records of 157 young AIS patients who were hospitalized in the Department of Neurology, Baoji Central Hospital, from February 2014 to October 2020. A signed informed consent form by themselves or their family members was obtained from all participants in this study, which was approved by the Medical Ethics Committee of Baoji Central Hospital (certificate of approval No. BJ2014D036). Written informed consent was obtained from the patients enrolled.

The inclusion criteria were as follows: (1) complying with the standards of the 2018 AIS diagnostic criteria, and a clear responsible lesion found by cranial computed tomography (CT) or magnetic resonance imaging (MRI) [9]; (2) reached hospital within 4 h from the onset of stroke before arterial recanalization; (3) the age of onset was 18–45 years; and (4) the NIHSS score was completed within 24 h of admission.

The exclusion criteria were as follows: (1) the first imaging revealed hemorrhagic cerebral infarction; (2) patients with neurological deficit symptoms; (3) patients with intracranial hemorrhage or intracranial tumor; (4) patients with severe heart, liver, and kidney diseases to avoid some complications; (5) patients taking lipid-lowering drugs in the past 3 months; and (6) patients with autoimmune or blood system diseases.

According to the results of the brain diffusion-weighted imaging (DWI) examination, the young AIS patients (observation group) were divided into HT (63 cases) and non-HT groups (94 cases) between February 2014 and October 2020. HT with a grade of HI1, HI2, PH1, or PH2 [European Cooperative Acute Stroke Study (ECASS)] was determined on the baseline or screening CT. All patients with AIS who were not thrombolytic within 7 d of onset were included. Head CT/MRI should be reviewed within 3 d of admission or when the condition worsens. The re-examination of head CT/MRI showed intracranial hemorrhage; all were ascertained to have HT. The data on gender, age, cerebral infarction-related risk factors, and other factors were collected. The comparison of PLR values and the relevant indicators between groups was mainly performed. PLR is calculated by dividing the absolute platelet count 10^9^/L by the absolute lymphocyte count ×10^9^/L.

### Evaluation of neurological damage

NIHSS rating was completed within 24 h after patient admission [10].

### Related hazardous factors diagnostic standards

The inclusion criteria included (1) time from onset to hospital admission within 24 h; (2) patients without thrombolysis; (3) first head CT/MRI to rule out intracranial hemorrhage; (4) patients with no history of stroke; (5) the age of onset was 18–45 years; and (6) the NIHSS score was completed within 24 h of admission. The exclusion criteria were as follows: (1) patients with lacunar infarction without neurological deficit symptoms; (2) patients with serious medical diseases, such as liver and kidney failure, heart failure, and tumors; (3) those who have not re-examined head CT/MRI during hospitalization; (4) previous history: hypertension (prestroke with a clear history of hypertension, antihypertensive medication, or at least two measurements of blood pressure >140/90 mmHg at least 1 week apart), diabetes (prestroke with a clear history of diabetes, insulin therapy, or oral hypoglycemic drugs or fasting blood sugar >7.0 mmol/L or random blood glucose >11.1 mmol/L or glycosylated hemoglobin >6.5%), hyperlipidemia (with a clear history of hyperlipidemia before stroke, lipid-lowering therapy, or blood cholesterol >6.22 mmol/L), history of atrial fibrillation (a clear history of atrial fibrillation before stroke, and history of stroke (previous diagnosis of any ischemic stroke or hemorrhagic stroke); and (5) patients taking lipid-lowering drugs in the past 3 months.

### Statistical processing

The SPSS 20.0 software was used for statistical analysis. Data were expressed as mean ± standard deviation (x ± s). The two groups were compared using independent samples with *t* test, whereas multiple groups were compared using the Student–Newman–Keuls *q* test. Non-normally distributed measurement data were analyzed using nonparametric test. Chi-square test was used for counting data. The Pearson's correlation coefficient was used for correlation analysis. The multivariate logistic regression analysis was used for further analyzing correlation factors, and Spearman's correlation coefficient was used for non-normally distributed data. The receiver operating curve (ROC) and area under the curve (AUC) were used to evaluate the predictive value of PLR in HT diagnosis in young AIS patients, and the difference was statistically significant with *P* < 0.05.

## Results

In this study, a total of 157 young patients with AIS were consecutively admitted to the hospital. The patients with cerebral infarction without thrombolysis within 7 d were divided into a non-HT group (94 cases, accounting for 59.87% of the total registration, with an average age of 35.85 ± 6.9 years) and HT group (63 cases, accounting for 40.13% of the registered number, with an average age of 33.75 ± 8.1 years). No significant difference was observed between the two groups of patients in terms of age, smoking status, gender, drinking, and hypertension (*P* > 0.05).

The basic clinical characteristics of 157 stroke patients, including age, gender, smoking, NIHSS score on admission, blood glucose, fibrinogen, blood lipid level, and PLR are shown in **Table 1**. The HT group was randomly assigned at the time of admission. The results showed that the PLR and NIHSS score were higher in the HT group than that in the non-HT group (**Table 1**).

**Table 1.** Comparison of baseline data between the observation group and control group

**Basic factors**	**Non-HT group (n = 94)**	**HT group (n = 63)**	**t/x^2^**	***P***
Sex (male, %)	59/60.8	39/61.9	0.003	0.958
Age (year)	35.85 ± 6.9	33.75 ± 8.1	0.372	0.666
Smoking, n (%)	24 (25.53)	16 (25.39)	0.116	0.142
Alcohol, n (%)	23 (23/93)	10 (10/63)	1.112	0.292
FBG (mmol/L)	7.427 ± 2.660	7.365 ± 3.131	0.139	0.1128
Atrial fibrillation	11 (11/93)	15 (15/63)	2.814	0.093
WBC (×109)	7.266 ± 2.547	7.237 ± 2.863	0.073	0.2543
PLT (×109)	279.2 ± 52.80	275.3 ± 49.89	0.4653	0.6423
NEUT (×109)	4.262 ± 0.8894	4.390 ± 0.8848	0.9808	0.374
LYM (×109)	1.254 ± 0.3528	1.311 ± 0.374	0.966	0.336
TC (mmol/L)	4.410 ± 0.4997	4.311 ± 0.7162	1.015	0.312
TG (mmol/L)	1.441 ± 0.2841	1.489 ± 0.2812	1.029	0.3052
LDL-C (mmol/L)	3.224 ± 0.8313	3.119 ± 0.498	0.903	0.368
HCY (μmol/L)	17.615 ± 4.2904	16.914 ± 5.4401	0.90	0.370
FIB (g/L)	3.163 ± 0.6877	3.382 ± 0.809	1.747	0.083
D-D (mg/L)	0.5832 ± 0.3270	0.2484 ± 0.07191	0.219	0.827
PLR	100.654 ± 47.667	139.219 ± 54.945	4.672	<0.0001
NIHSS	9.0745 ± 4.467	10.667 ± 4.276	2.226	0.027

D-D, D-dimer; FBG, fasting blood glucose; FIB, fibrinogen; HCY, homocysteine; LYM, lymphocyte; NEUT, neutrophil; NIHSS, National Institute of Health Stroke Scale; PLR, platelet to lymphocyte ratio; PLT, platelet; TC, total cholesterol; TG, triglycerides; WBC, white blood cell.

### HT risk factor

Subsequently, the factors listed in **Table 1** were analyzed using univariate logistic regression.

**Table 2** shows that the PLR and NIHSS were risk factors of HT and illustrated the relationship between clinical characteristics and HT. NIHSS and PLR were associated with HT. In the multivariate analysis, PLR and NIHSS reached significance in the HT groups (**Table 2**).

**Table 2.** Univariate and multivariate Logistic Regression Analysis for Factors Associated with hemorrhagic transformation

**Variables**	**Univariate analysis**			**Multivariate analysis**		
	
	**B (beta values)**	**P**	**95% CI (Confidence interval)**	**B (beta values)**	**P**	**95% CI (Confidence interval)**
PLR	0.14	<0.0001	0.979–0.992	0.31	<0.0001	0.956–0.984
NIHSS	0.082	0.029	6.032	0.202	0.014	1.042–1.438

### The correlation of NIHSS score with PLR

**Figure 1.**
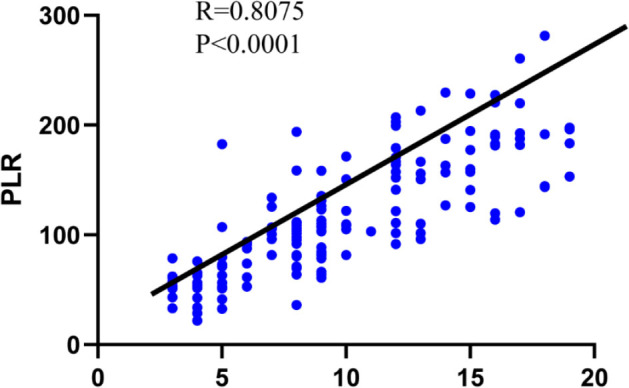
Pearson's correlation of the PLR levels and NIHSS score.

Pearson's correlation showed a positive correlation between the PLR and NIHSS score (**Figure 1**).

### Receiver operating curve analysis results

**Figure 2.**
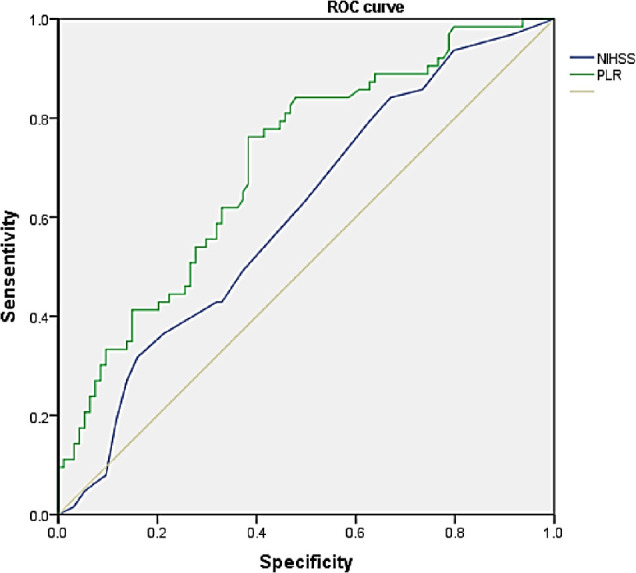
The ROC curve of PLR levels and NIHSS.

The role of PLR in HT diagnosis in young AIS patients was evaluated by ROC analysis. ROC analysis revealed that PLR can predict HT (ROC area, 0.708; *P* < 0.0001) with more sensitivity and specificity than NIHSS (ROC area, 0.607; *P* < 0.0001; **Figure 2**).

## Discussion

The strengths of this study were as follows: the PLR value was associated with the NIHSS score after HT in young AIS patients; the change in the value may reflect the severity of hemorrhage. In the clinical work of the neurology department, the PLR value can be considered as a predictive index for assessing the occurrence of HT in young ACI patients as early as possible, and it can also be used as an effective index for assessing the severity of HT, which provides a basis for the further guidance of clinical treatment. To the best of our knowledge, this is the first study to evaluate the association between PLR and HT in young AIS patients.

With the continuous increase in social development, the incidence of young AIS patients is increasing year by year [1], as young people bear more social responsibilities. Thus, the effect of young AIS on the family and society is quite obvious [11]. Studies have shown that the main cause of young AIS is atherosclerosis [12]; however, hypertension, type 2 diabetes, hyperlipidemia, smoking, and alcohol are still the main risk factors for cerebral arteriosclerosis in young people [13,14,15]. The results of this study showed that the main risk factors for HT patients were atrial fibrillation and NIHSS, which are consistent with the results of previous studies.

Studies have shown that after AIS, acute ischemia and hypoxia occur in the local brain tissue due to infarction, causing platelets to release inflammatory molecules [5] and enhancing the process of vascular inflammation. This will thus affect the severity and prognosis of cerebral infarction [16, 17].

Lymphocytes are related to the body's own immunity and represent cellular and humoral immunity in the body. When acute inflammation occurs, lymphocyte levels can be easily decreased [18]. PLR is a new type of inflammatory marker that is easily available and reproducible [7]. The higher the PLR, the more severe the inflammatory response [19]. Previous studies have reported that increased PLR will increase the risk of arterial plaque shedding [20] and is associated with cerebral infarction volume and poor prognosis [8]. The increase in the PLR value may be because cerebral ischemia and hypoxia after AIS increase the release of platelets, leading to the release of a series of inflammatory factors into the blood. Therefore, the lymphocyte level that provides autoimmune protection decreases. Inflammatory factors interacted with platelets and lymphocytes, and the vascular inflammatory response was enhanced, resulting in a significant increase in the PLR value. However, the exact pathological mechanism remains unclear, and further basic and clinical studies are warranted. PLR values were closely associated with the onset of HT in young AIS patients, indicating that PLR values can be used as a new type of inflammation index and for early screening of young AIS patients. Therefore, an effective inflammation prevention measure can be considered early to reduce the occurrence of HT and other poor prognostic diseases in young AIS patients. It shows more positive clinical significance.

In this study, the correlation between NIHSS scores and PLR was analyzed. The results showed that the PLR value was positivity correlated with NIHSS scores (*r* = 0.8075, *P* < 0.0001). The PLR value increased as the nerve damage worsened, which may be because of the more severe cerebral ischemia and hypoxia in patients with young AIS, and it strengthened the vascular inflammation. Thus, as an increased number of the inflammatory factors were released in the body, the release of platelets increased, and lymphocytes decreased, with a significant increase in PLR. Further studies are warranted to confirm the specific mechanism.

HT refers to secondary hemorrhage after AIS, which has been divided into hemorrhagic infarction and parenchymal hematoma by the European Cooperative Acute Stroke Study [21]. The occurrence of HT is associated with BBB destruction, reperfusion injury, poor collateral circulation, and other factors [22, 23]. Previous studies have suggested that the risk factors for HT include low platelets, abnormal coagulation function, advanced age, thrombolysis, hypertension, and diabetes [24, 25]. In this study, no significant difference was found in sex, smoking, alcohol, and hypertension between the HT and non-HT groups. We found a significant difference in PLR and NIHSS among patients with the HT group. Pearson's correlation analysis revealed that the PLR value was positively correlated with the NIHSS score in patients. As NIHSS increased, the PLR value also increased. Numerous studies have shown that the size of the infarct is a crucial factor in causing HT. Kerenyi et al. [26] found that the infarct size in patients with AIS was positively correlated with HT. In addition, Kablau et al. [27] proposed that large-scale cerebral infarction and severe NIHSS score at admission were risk factors for HT. This may be because after HT occurs in patients with AIS, vascular inflammation and edema manifest more obviously than simple infarction. Lymphocytes significantly decreased because of the immunopathological process. Although platelets temporarily decreased because of bleeding, with the prolongation of bleeding time, the release of inflammatory mediators increased significantly in the body, which in turn increased platelets and eventually led to a significantly higher PLR value than before HT. Therefore, the patients with higher PLR should accept that contrast-enhanced CT presents high diagnostic accuracy for the detection of renal hemorrhage.

The exact mechanism remains unclear, and further research is warranted. The ROC curve analysis showed that the AUC of PLR was 0.708, and the sensitivity and specificity were 0.806 and 0.674, respectively. The diagnostic value is worthy for HT in young acute cerebral infarction.

However, this study has several limitations. First, follow-up with the selected patients was not performed. The relationship between the patient's HT and PLR values cannot be completely evaluated because of the lack of understanding of the dynamic evolution process. Second, blood routine was susceptible to multiple assistant factors, and the value fluctuated greatly. Therefore, the influence of the above factors on the results of this study cannot be overlooked. Therefore, multiple tests are required, and the calculated average value may increase the accuracy. Third, this study was a single-center retrospective study of medical records, including a small number of patients. The exact mechanism between the PLR value and the occurrence of HT in young AIS patients remains unknown. Additionally, there were some concerns due to the sensitivity and specificity of CT/MRI to detect HT. Therefore, large-scale basic research is still needed.

Conclusively, in young AIS patients, an increase in PLR value can predict the risk of HT. According to PLR value, it is critically important for AIS patients who are still young to take proactive steps to prevent HT.
